# Host and Viral Factors Involved in Nuclear Egress of Herpes Simplex Virus 1

**DOI:** 10.3390/v13050754

**Published:** 2021-04-25

**Authors:** Jun Arii

**Affiliations:** Division of Clinical Virology, Center for Infectious Diseases, Kobe University Graduate School of Medicine, Kobe, Hyogo 650-0017, Japan; jarii@med.kobe-u.ac.jp; Tel.: +81-78-382-6272

**Keywords:** herpesviruses, nuclear egress, primary envelopment, de-envelopment

## Abstract

Herpes simplex virus 1 (HSV-1) replicates its genome and packages it into capsids within the nucleus. HSV-1 has evolved a complex mechanism of nuclear egress whereby nascent capsids bud on the inner nuclear membrane to form perinuclear virions that subsequently fuse with the outer nuclear membrane, releasing capsids into the cytosol. The viral-encoded nuclear egress complex (NEC) plays a crucial role in this vesicle-mediated nucleocytoplasmic transport. Nevertheless, similar system mediates the movement of other cellular macromolecular complexes in normal cells. Therefore, HSV-1 may utilize viral proteins to hijack the cellular machinery in order to facilitate capsid transport. However, little is known about the molecular mechanisms underlying this phenomenon. This review summarizes our current understanding of the cellular and viral factors involved in the nuclear egress of HSV-1 capsids.

## 1. Introduction

**Figure 1:**
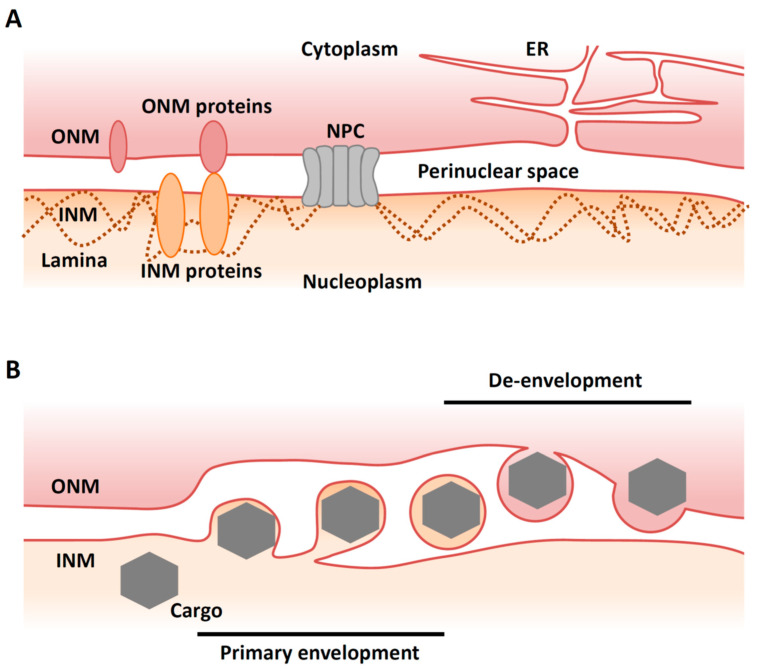
Overview of nuclear egress. (**A**) Illustration of a ‘normal’ nuclear envelope. INM, inner nuclear membrane; ONM, outer nuclear membrane; NPC, nuclear pore complex; ER, endoplasmic reticulum. (**B**) Illustration of vesicle-mediated nucleocytoplasmic transport. A cargo (e.g., viral capsid) in the nucleus buds through the INM to form a vesicle in the perinuclear space that then fuses with the ONM to release the complex into the cytoplasm (de-envelopment).

The nuclear membrane (NM) consists of an inner nuclear membrane (INM) and an outer nuclear membrane (ONM), which separate nuclear and cytoplasmic activities in the eukaryotic cell (Figure 1A). The segregation of the nucleoplasm from the cytoplasm separates translation from transcription and maintains genome integrity. The INM and ONM are biochemically distinct [1]. While the ONM is continuous and functionally interacts with the endoplasmic reticulum (ER), the INM contains its own set of integral membrane proteins [2]. The ONM and the INM are separated by the perinuclear space and connected at annular junctions, where nuclear pore complexes (NPCs) are found. NPCs are large macromolecular assemblies that form transport channels and regulate trafficking between the cytoplasm and the nucleoplasm [3]. It had long been assumed that the NPC was the only route out of the nucleus except during the early phases of mitosis when the nuclear envelope breaks down.

Recently, however, an alternative and NPC-independent pathway for nuclear export has been described in cells infected with viruses. Vesicle-mediated nucleocytoplasmic transport is a mechanism for the nuclear export of macromolecular complexes [4]. In this system, a macromolecular complex in the nucleus buds through the INM to form a vesicle in the perinuclear space (‘primary envelopment’). This vesicle then fuses with the ONM to release the complex into the cytoplasm in a process called ‘de-envelopment’ [4] (Figure 1B). This type of transport is observed in herpesvirus-infected mammalian cells and is required for the nuclear export of viral capsids that assemble in the nucleus [5]. Nucleocytoplasmic transport (or ‘nuclear egress’) of capsids is essential for the life cycle of all herpesviruses, as final maturation of their virions occurs in the cytoplasm. Although vesicle-mediated nucleocytoplasmic transport is not common in uninfected mammalian cells, it has been reported that *Drosophila* cellular large ribonucleoprotein complexes (RNPs) utilize this nuclear export mechanism in the absence of viral infection [6].

The goal of this review is to present the current knowledge of the cellular factors that are involved in vesicle-mediated nucleocytoplasmic transport of herpesvirus capsids. In particular, the review focuses on herpes simplex virus 1 (HSV-1), which has been extensively studied and for which the mechanisms of capsid nuclear egress are relatively well understood. The author hopes that the review will stimulate researchers in the field to engage in new research projects that will further elucidate the many mysteries of this transport system.

## 2. Overview of Nuclear Egress of Herpesviruses

Herpesviruses are enveloped double-stranded DNA viruses that establish lifelong latent infections in their natural hosts [7]. The *Herpesviridae* family is subdivided into the *Alphaherpesvirinae, Betaherpesvirinae*, and *Gammaherpesvirinae* subfamilies, based on their molecular and biological properties [7]. HSV-1 is the prototype of the alphaherpesvirus subfamily, which is comprised of the most common pathogenic agents in humans, and causes a variety of conditions such as mucocutaneous disease, keratitis, skin disease and encephalitis [8]. Herpesviruses share a common virion structure and similar proliferation strategies. A mature virion consists of an icosahedral capsid with a linear double-stranded DNA genome, a proteinaceous layer called tegument, and a host-membrane derived envelope spiked with viral glycoproteins.

Herpesviruses replicate their genomes and package them into capsids within the host cell nucleus. These capsids must traverse from the nucleus to the cytoplasm through a process called nuclear egress. In brief, the nascent nucleocapsids bud at the INM to form primary virions in the perinuclear space, and then the envelopes of primary virions fuse with the ONM, releasing the nucleocapsids into the cytoplasm for further maturation (Figure 1B). In the cytoplasm, nucleocapsids bud into vesicles derived from trans-Golgi networks or endosomes in a process called ‘secondary envelopment’. Finally, enveloped virions are released from the cells through an exocytotic pathway [5].

This envelopment/de-envelopment model of herpesvirus egress is now generally accepted and appears to be the dominant mechanism employed by this virus. However, other routes have been proposed. For example, capsids may be transported concomitantly with nuclear envelope breakdown in certain situations [9]. It has also been reported that capsids may egress from the nucleus through defective nuclear pores [10,11].

## 3. The Nuclear Egress Complex (NEC)

The NEC is composed of the conserved viral proteins UL31 and UL34 and plays a crucial role in the nucleocytoplasmic transport of newly assembled nucleocapsids [5,12]. In the absence of either protein, capsids do not bud at the INM and instead accumulate inside the nucleus [13,14,15,16,17,18]. UL34 is a type II integral membrane protein that is targeted to both the INM and the ONM [19,20]. UL31 is a soluble nuclear protein that is held in close apposition to the inner and outer surfaces of the INM and ONM through its interaction with UL34 [20]. Conversely, INM localization of UL34 is enhanced in the presence of UL31 [16,20]. Crystal structures of NEC from HSV-1 and the other members of *Herpesviridae* reveal the highly conserved features of this complex [21,22,23,24,25].

## 4. Primary Envelopment

### 4.1. Lamina Dissociation

Nuclear shape and mechanical stability are maintained by the nuclear lamina, which is localized beneath the INM. This structure is primarily composed of lamins, which are members of the intermediate filament family of cytoskeletal proteins (Figure 1A). Lamins interact with a large number of proteins at the INM and in the nucleoplasm, thereby influencing structural stability of the nucleus, DNA replication, transcription, and chromatin remodeling [26].

**Figure 2:**
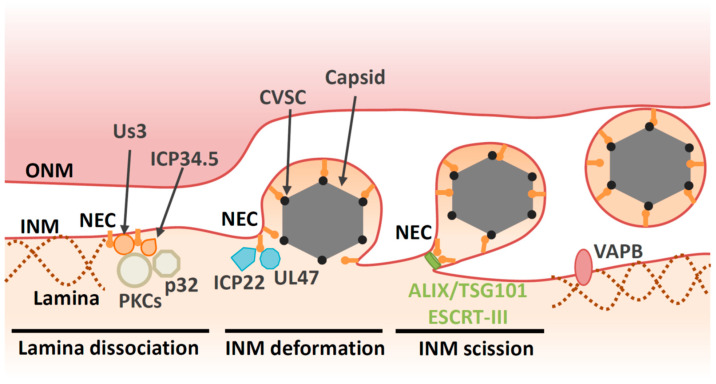
Multiple proteins are involved in primary envelopment of HSV-1 capsids. NEC and kinases (e.g., PKCs and Us3) induce lamina dissociation to allow capsid recruitment to the INM. ICP34.5 and p32 contribute to the recruitment of PKCs. The NEC is responsible for capsid recruitment and mediates budding by assembling into a hexagonal lattice. CVSC decorates vertices of DNA-filled nucleocapsid and promotes nuclear egress through interaction with the NEC. Viral proteins (e.g., UL47 and ICP22) and cellular protein VAPB regulate primary envelopment through as-yet unidentified mechanisms. The NEC recruits ESCRT-III via ALIX to pinch off primary virions from the INM.

Before HSV-1 capsids access the INM, the nuclear lamina meshwork has to be dissolved. Between prophase and metaphase of mitosis, the lamina disassembles before reassembling during interphase [1]. This lamina disassembly is tightly regulated by phosphorylation [1]. During herpesvirus infection, the lamina is disrupted locally [27,28,29,30,31] (Figure 2). It appears that the NEC component UL34 recruits protein kinase C (PKC) isoforms that phosphorylate lamin B during infection [27,32]. In addition, viral kinases are also required for lamina dissolution. Viral kinase Us3, which is conserved in alphaherpesviruses, phosphorylates the lamin A/C protein (LMNA) [33,34,35]. HSV-1 encodes the other kinase UL13, which is conserved in *Herpesviridae*. UL13 homologues found in *Betaherpesvirinae* and *Gammaherpesvirinae* have predominant roles in lamin dissociation [36,37,38]. However, this function of UL13 homologues is not conserved in HSV-1 UL13 [36].

Furthermore, the NEC binds directly to LMNA to modulate its conformation [29] and perturbs the interaction of lamins with emerin, a protein that links the lamina to the INM [39,40]. Taken together, the NEC directly and indirectly disrupts the fibrillar network of the nuclear lamina to ensure correct docking of capsids to the INM. Viral proteins also regulate cellular components beyond the NEC in order to facilitate capsid egress. For example, the HSV-1 neurovirulence factor ICP34.5 also contributes to lamin dissociation through recruitment of PKC to the NM via the cellular protein p32 [41,42] (Figure 2).

New data have underscored the importance of the lamin meshwork as a barrier to viral replication. Lamin degradation is required for HSV-1 nuclear egress and growth in dendritic cells [43]. Tripartite Motif Containing (TRIM) 43 is a factor that restricts a broad range of herpesviruses and is upregulated during viral infection [44]. Interestingly, TRIM43 mediates degradation of the centrosomal protein, pericentrin, which subsequently leads to alterations of the nuclear lamina. This alteration suppresses transcriptionally active viral chromatin states and viral replication.

In general, lamins contribute to the regulation of chromatin organization by tethering peripheral heterochromatin and chromatin remodeling complexes to the nuclear envelope [26]. Evidence of LMNA’s role in chromatin organization is provided by the finding that mutations in the human *LMNA* gene lead to premature aging and progressive loss of heterochromatin [45]. The same is true for the HSV-1 genome, as epigenetic regulation of viral DNA is modulated by its binding to LMNA [46,47,48]. These observations suggest that viral gene expression and nuclear egress must proceed in a coordinated manner.

### 4.2. Deformation of the INM

In addition to the disintegration of lamins, the NEC is responsible for recruitment of capsids to the INM [13,14,49,50]. Capsid vertex-specific component (CVSC), which consists of the UL17 and UL25 proteins, decorates vertices of DNA-filled nucleocapsids [51,52,53,54]. CVSC is proposed to promote nuclear egress of DNA-filled nucleocapsids through interaction with the NEC [55,56,57,58,59]. Once the capsid is recruited to the INM, primary envelopment occurs. This process involves membrane deformation around the capsid, followed by a ‘pinching off’ of the nascent bud; the resulting vesicle is formed in the perinuclear space and is referred to as the ‘primary virion’ (Figure 2). The NEC plays a direct role in nucleocapsid budding at the INM. Transient coexpression of NEC from pseudorabiesvirus (PRV, a porcine alphaherpesvirus) or Kaposi’s sarcoma-associated herpesvirus (KSHV, a human gammaherpesvirus) induces formation of perinuclear vesicles [60,61,62]. Moreover, recombinant NEC from HSV-1 or PRV can promote vesiculation of synthetic lipid membranes in the absence of any other viral or cellular proteins [63,64]. These findings suggest that the NEC has an intrinsic ability to deform membranes. Disruption of the membrane-coupled hexagonal NEC lattice by mutation of the inter- or intrahexagonal contact site impairs vesiculation both in vitro and in infected cells [4,21,49,63,65]. Thus, it is likely that formation of the NEC lattice drives vesiculation [4,21,63].

Biochemical analysis of the NEC also shows the requirement for a specific lipid composition for vesicle formation in vitro. Although the role of lipid in infected cells has not been analyzed, a high concentration of phosphatidic acid (PA) is required for in vitro vesicle formation [63]. Recently, it has been reported that PA-rich membranes at nuclear envelope herniation sites recruit cellular membrane remodeling machinery to the NMs in budding yeast [66]. Cell membranes mainly consist of lipids and thus lipid composition must affect the viral entry and the process by which capsid vesicles form during the budding processes. Protein kinase D (PKD) regulates vesicle transport through phosphorylation of phosphatidylinositol 4-kinase β (PI4KIIIKβ), which converts phosphoinositide (PI) to phosphoinositol 4-phosphate (PI4P). Knockdown of PKD or its modulators impairs both primary envelopment and virion release [67], suggesting that lipid composition plays a role during primary envelopment. Phosphatidylethanolamine (PE) a phospholipid that plays significant roles in cellular processes, such as vesicle transport and membrane fusion. Inhibition of the PE synthesis pathway impairs envelopment of HSV-1 capsids at the cytoplasm, but has no effect on nuclear egress [68]. The exact composition of specific lipids and the requirement for these during primary envelopment in infected cells should be determined during future studies.

### 4.3. Factors Involved in INM Deformation

Although the NEC has ability to vesiculate membranes in transfected cells and in vitro [4,21,63], empty perinuclear vesicles are rarely observed during infection. Therefore, in infected cells, the intrinsic budding potential of the NEC has to be regulated tightly before capsids arrive to the INM. In other words, NEC activity alone may not be sufficient for primary envelopment in infected cells where the environment is more complex.

Indeed, several other viral factors are reported to contribute nuclear egress in infected cells (Table 1). Tegument is a layer comprising thousands of densely packaged proteins that are localized between the virion envelope and capsid. The assembly of tegument on capsids occurs predominantly in the cytoplasm following nuclear egress. However, accumulating evidence suggests that capsids acquire some of the tegument proteins in the nucleus. In particular, tegument proteins UL36 (VP1/2), UL37, UL41 (vhs), UL47 (VP13/14) UL48 (VP16), UL49 (VP22), Us3, ICP0 and ICP4 have been reported to localize in the nucleus or associate with capsids [69,70,71,72,73]. While UL36 is not essential for nuclear egress of HSV-1 or PRV, a nuclear-specific isoform comprising the C-terminal region of UL36 is recruited to PRV capsids in the nucleus and may enhance their nuclear egress [74,75]. Cryo-EM analyses reveal that UL36 forms part of the HSV-1 CVSC, which was originally considered to be a complex of the UL17 and UL25 proteins [76,77]. Contributions of other HSV-1 proteins on primary envelopment have been described. UL47, the major tegument protein of HSV-1, interacts with the NEC and promotes primary envelopment [78]. The immediate early protein ICP22, which is conserved in the subfamily *Alphaherpesvirinae,* interacts with the NEC and regulates NEC localization and HSV-1 primary envelopment [79] (Figure 2).

In addition to the viral factors described above, host cell factors also contribute to vesicle formation at the INM during primary envelopment through as-yet undefined mechanisms. Vesicle-associated membrane protein-associated protein B (VAPB), which mediates cytoplasmic vesicle transport at the ER, facilitates HSV-1 nuclear egress [94]. Viral and cellular factors involved in HSV-1 primary envelopment are listed in Table 1 and Table 2, respectively.

### 4.4. Scission at the INM during Primary Envelopment

Although NECs have the intrinsic capability to remodel membranes, they are unable to mediate primary envelopment by themselves in infected cells, as described above. It appears that host endosomal sorting complexes required for transport (ESCRT)-III machinery is responsible for the scission step during HSV-1 primary envelopment [80]. Viral budding can be divided into two stages: membrane deformation (the membrane is wrapped around the assembling virion) and membrane scission (the bud neck is severed) (Figure 2). A remarkable variety of enveloped viruses appear to use host ESCRT-III machinery at the scission stage of budding [95]. The ESCRT-III proteins were initially discovered as factors that are required for the biogenesis of multivesicular bodies (MVBs). It is now clear that the functions of the ESCRT-III proteins extend far beyond their role in MVB formation. Indeed, ESCRT complexes also regulate cytokinesis, the biogenesis of microvesicles and exosomes, plasma membrane repair, neuron pruning, the quality control of NPC, nuclear envelope reformation, and autophagy [96]. ESCRT-III proteins exist as soluble monomers in solution. When activated by membrane-associated upstream factors such as ESCRT-I/II, ALG-2-interacting protein X (ALIX) or charged multivesicular body protein (CHMP)7, ESCRT-III assembles into filamentous structures on membranes. These ESCRT-III assemblies are thought to be the main drivers of membrane remodeling and scission. The hexameric AAA ATPase Vps4 drives remodeling and disassembly of ESCRT-III filaments, and inactivation of Vps4 severely impairs ESCRT-III-mediated membrane remodeling [96].

Herpesvirus replication appears to rely on the ESCRT-III machinery [97,98,99,100]. At first, HSV-1 nuclear egress was thought to be independent of the ESCRT-III machinery in HEK293 cells as it is insensitive to a dominant negative mutation of Vps4 [97]. However, depletion of an ESCRT-III protein or its adaptor, ALIX in HeLa cells, reveals the role of ESCRT-III machinery during nuclear egress of HSV-1 [80]. In these cells, viral budding is arrested at the lollipop stage and nearly complete immature virions remained tethered to the INM. These nearly complete virions were morphologically similar to the virions of other viruses that are arrested during budding in the absence of ESCRT factors [95]. Furthermore, ESCRT-III is recruited by the NEC to the INM during the nuclear egress of HSV-1 capsids [80]. ESCRT-II, ALIX and CHMP7 have been identified as upstream factors of ESCRT-III that recruit ESCRT-III proteins and initiate their assembly [96]. In these contexts, CHMP7, but not ESCRT-II or ALIX, is required for NM reformation [101,102]. However, ALIX is required for the scission in the INM during nuclear egress [80] (Figure 2). In agreement with this observation, ALIX is required for recruitment of ESCRT-III to the NM during infection of cells with Epstein–Barr virus (EBV, a human gammaherpesvirus) [103,104]. In addition, a compound that binds to ESCRT-I protein TSG101 inhibits primary envelopment of HSV-1 in Vero cells [105], supporting the idea that ESCRT-III machinery is required for nuclear egress of HSV-1 capsids. The other ESCRT-III adaptor CHMP7 has a predominant role in reformation of the nuclear membrane in various situations [101,102]. Thus, it is unlikely that ESCRT-III is recruited to repair HSV-1-induced defects in the nuclear envelope. Taken together, these data suggest that, while NEC has ability to promote vesiculation in vitro, it recruits ESCRT-III machinery to complete scission in infected cells. Similarly, Ebola virus VP40 alone can carry out membrane budding in an in vitro system [106], although Ebola virus budding is highly dependent on ESCRT-III machinery [95].

## 5. De-Envelopment

### 5.1. Overview of De-Envelopment

De-envelopment is a process in which perinuclear virions fuse with the ONM to release naked capsids into the cytosol (Figure 1B). Membrane fusion and disassembly of the NEC are required in this process. The molecular mechanism of de-envelopment is not well understood. Nonetheless, several viral and cellular proteins have been reported as regulators of HSV-1 de-envelopment (Table 1 and Table 2).

**Figure 3:**
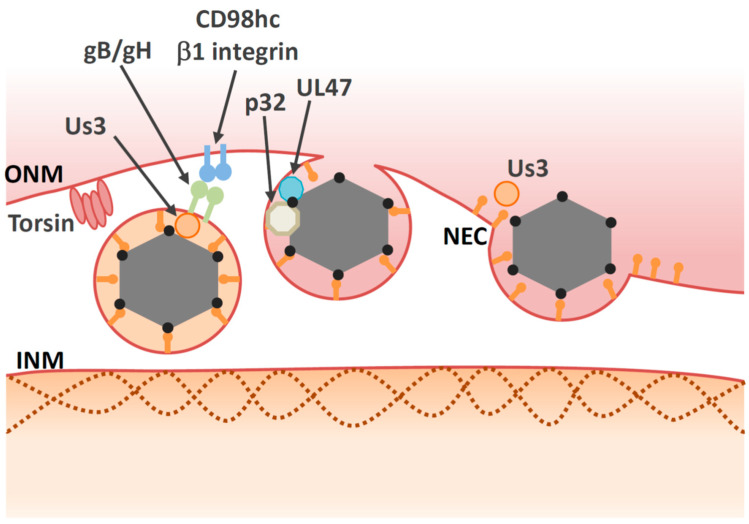
Multiple proteins are involved in the de-envelopment step. Some viral proteins, such as gB/gH and Us3 may be involved in the de-envelopment step, but their roles are unclear. Us3 may induce disassembly of the NEC lattice. Torsin and p32 promote de-envelopment through unidentified mechanisms. Viral protein UL47 interacts with cellular protein p32. The CD98hc/β1 integrin complex may influence fusion activity of the primary virions.

In particular, the Us3 protein kinase, which is conserved among *Alphaherpesvirinae*, has a prominent role in the de-envelopment step. Inactivation of Us3 enzymatic activity leads to an accumulation of primary enveloped HSV-1 virions in large invaginations of the INM [83,107,108], indicating that phosphorylation may regulate the de-envelopment process, even though it is not essential. The NEC lattices within the perinuclear virions are stable structures that must be disassembled during de-envelopment. Phosphorylation of the NEC by Us3 may loosen NEC lattices in perinuclear virions to promote de-envelopment [81,87] (Figure 3). In agreement with this hypothesis, Us3 is present in perinuclear virions [83]. As is the case with Us3 kinase inactive mutants, mutations in other viral tegument protein UL51 of HSV-1 also lead to accumulation of primary envelope virions in the perinuclear space [92,109,110], suggesting that the de-envelopment process is modulated by various viral proteins.

Similar to membrane fusion during virus entry, virion glycoproteins may play roles in mediating fusion between the envelope of the primary virions and the ONM. Primary virions accumulate in the perinuclear space during infection with recombinant HSV-1s harboring null or point mutations in both gB and gH [85,90,111]. To date, it is unclear how either gB or gH mediate fusion during de-envelopment, although both are required for fusion during viral entry [112] and Us3 phosphorylates gB [85,86]. In the case of the closely related PRV protein, gB or gH are not present in the perinuclear vesicles or the NMs and they do not have any role on nuclear egress [91]. Thus, gB or gH/gL do not appear to function as conserved components of the fusion machinery during de-envelopment step.

Deletion of either UL31 or UL34 completely halts perinuclear virion formation, which occurs before de-envelopment. Thus, it is conceivable that UL31 and/or UL34 also play an essential role in the de-envelopment step as well as in primary envelopment. UL34 is a transmembrane protein, the C-terminus of which is exposed to the perinuclear space. However, the C-terminal domain (including the transmembrane region) can be replaced by corresponding regions of other viral or cellular proteins without loss of function [113,114]. These observations indicate that the NEC does not directly play a role in the fusion process and that other components of the cellular fusion machinery are involved in de-envelopment. Thus, viral proteins that are involved in the de-envelopment process described above (Table 1) might also modulate cellular fusion machinery at the ONM.

### 5.2. Cellular Factors Involved in De-Envelopment

In addition to viral factors, cellular factors also regulate the de-envelopment process (Figure 3 and Table 2). Cellular factors including p32, CD98 heavy chain (CD98hc) and β1 integrin are recruited to the NM in HSV-1-infected cells [115,116]. Knockdown of these proteins leads to aberrant accumulation of enveloped virions in the invagination structures derived from the INM; this is phenocopied by inactivation of Us3 protein kinase activity. These observations suggest that the cellular factors mentioned above regulate the de-envelopment step. Accumulation of the multifunctional scaffold protein, p32, at the nuclear rim in infected cells depends on UL47 [116], suggesting that UL47 has an optimal role in the de-envelopment step in addition to its role in primary envelopment [78]. Plasma membrane protein CD98hc may regulate membrane fusion, since anti-CD98hc monoclonal antibodies enhance or inhibit cell–cell fusion, mediated by fusion proteins [117,118,119,120]. This CD98hc function in membrane fusion involves its binding partner, β1 integrin [119], suggesting that the CD98hc/β1 integrin interaction regulates fusion between perinuclear virions and the ONM. In the absence of UL34, CD98hc is not redistributed to the NM, but is dispersed throughout the cytoplasm [121]. At the ultrastructural level, HSV-1 infection causes ER compression around the nuclear envelope, whereas the UL34-null mutation causes cytoplasmic dispersion of the ER [121]. These observations suggest that HSV-1 infection redistributes ER around the nuclear envelope through unidentified mechanisms, thereby enabling accumulation of ER-associated de-envelopment factors, such as CD98hc, gB, and gH in the region where de-envelopment occurs.

Several lines of evidence indicate that torsins might be involved in HSV-1 nuclear egress. Torsins are members of the AAA+ ATPase superfamily, which is comprised of enzymes that mediate ATP-dependent conformational remodeling of target proteins or protein complexes [125]. Among torsin orthologues in humans, Torsin A (TorA) is the best studied due to its association with a disease designated as early onset dystonia; this association underlies the alternate name for this protein, which is dystonia 1 protein (DYT1) [125]. Torsins are located within the lumen of the ER and NM [126]. In contrast to other AAA+ ATPases, torsins lack a key catalytic residue and need the INM protein lamina-associated polypeptide 1 (LAP1) or the ER protein lumenal domain like LAP1 (LULL1) as a cofactor for full activation [127,128]. Deletion or mutation of TorA resulted in the accumulation of vesicles in the perinuclear space [126]; these vesicles resembled those produced in cells expressing NEC [60]. Ectopic expression of wild-type TorA reduced HSV-1 production and led to accumulation of virus-like structures in the perinuclear space and the lumen of the ER [123], indicating that torsins play a role in de-envelopment. Similarly, perinuclear virions accumulate in TorA-mutant murine cells infected with HSV-1 [129]. Although torsins are involved in nuclear egress, the precise molecular mechanisms by which they regulate this process remain unclear. Another study showed that knockout of torsin itself or the INM-specific torsin cofactor, LAP1, produced only subtle defects in viral replication [130]. In this study, the authors also showed that knockout of LULL1, a torsin cofactor in the ER, reduces HSV-1 growth by one order of magnitude in the absence of nuclear egress defects. Taken together, torsins may be involved in HSV-1 nuclear egress indirectly, as they appear to regulate the nuclear envelope [131,132] and lipid metabolism [133], and both these biological processes have an impact on nuclear egress. Although torsins have been extensively investigated in biochemical studies and animal models, their precise biological functions remain elusive. Further study of their roles in the nuclear egress of herpesvirus might help elucidate these functions.

Neither the de-envelopment step nor viral replication are completely dependent on any of the individual cellular factors described above (Table 2). This suggests a degree of redundancy among these factors, which presents a challenge when attempting to characterize the precise contribution of each. Future biochemical analyses or fusion assays designed to reconstitute the de-envelopment step are desirable in this regard, as they will help to elucidate molecular mechanisms. Alternatively, it is possible that the major cellular players in vesicle-mediated nuclear egress have not been identified yet. Notably, there have been no reports to date of an a ONM-resident factor that is involved in fusion.

In general, an entry receptor is required for viral glycoprotein-driven fusion. The entry receptor restricts susceptible cells and determines the tropism of a particular virus. A receptor-like molecule that increases affinity for the perinuclear virion would therefore restrict the ‘perinuclear tropism’, thereby preventing back-fusion to the INM. If viral fusion protein gB mediates fusion between the perinuclear virion and ONM [85,90,111], it might also associate with membrane proteins in the ONM, as gB receptors are important during viral entry [134,135,136]. As described above, however, gB is not essential for the de-envelopment step. Thus, both a ‘ligand’ in the perinuclear virion and a ‘receptor’ in the ONM must be identified to prove this hypothesis.

## 6. Vesicle-Mediated Nucleocytoplasmic Transport in Uninfected Cells

**Figure 4:**
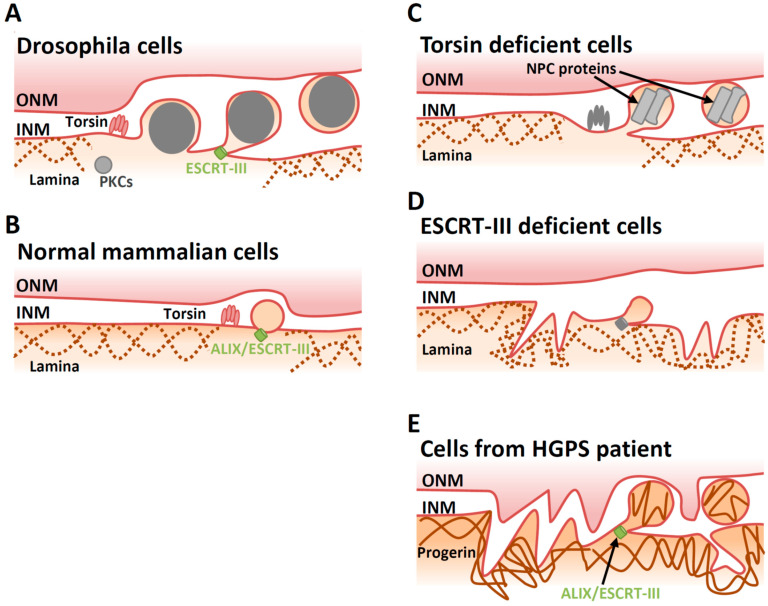
Proposed models of vesicle-mediated cytoplasmic transport in various situations. (**A**) A large RNP complex exits from the nucleus through vesicle-mediated nucleocytoplasmic transport system in *Drosophila* cells. (**B**) In ‘normal’ mammalian cells, torsins and ESCRT-III might contribute to eliminate excess INM and/or INM proteins. This process might be similar to the vesicle-mediated nucleocytoplasmic transport of HSV-1 nucleocapsids. (**C**) In torsin-deficient cells, herniations and vesicles containing NPC proteins within the perinuclear space are produced. (**D**) In ESCRT-III-deficient cells, INM proliferation is induced. (**E**) In cells from HGPS patients, progerin (mutant lamin A) accumulates in the nucleus, resulting in nuclear envelope deformation and defects including vesicles in the perinuclear space. ESCRT-III represses membrane deformation in these cells.

For a long time, vesicle-mediated nucleocytoplasmic transport was considered to be unique to the infection process used by herpesviruses. However, *Drosophila* RNPs also utilize this nuclear export mechanism [6] (Figure 4A). Interestingly, formation of perinuclear RNPs requires PKC-dependent lamin phosphorylation; parallels can be drawn with the phosphorylation of lamins that occurs during nuclear egress of herpesvirus capsids [6]. Moreover, torsin, lamin and ESCRT-III are important for nucleocytoplasmic transport of *Drosophila* RNPs, which is again similar to the requirements of viral nuclear egress [80,122,124]. These observations indicate that herpesviruses may have expropriated this transport mechanism.

Although perinuclear large vesicles are rarely seen in normal mammalian cells, vesicle-mediated cytoplasmic transport might occur in them. Mutation in torsin produces omega-shaped herniations and vesicles containing NPC proteins within the perinuclear space in murine and human cells [131,132,137]. Thus, torsins may regulate the vesicle-mediated cytoplasmic transport system in normal cells, and impairment of torsins leads to the accumulation of aberrant intermediate structures in the perinuclear space [125,126] (Figure 4B,C). Similarly, aberrant perinuclear blebs and vesicles are observed in ESCRT-III-deficient yeast cells, as ESCRT-III has the ability to clear NPC assembly intermediates [138,139]. In mammalian cells, ESCRT-III is involved in resealing the NM during late anaphase [101,140] and in repairing NM ruptures caused by cell migration through tight interstitial spaces [102,141]. In addition to these effects, impairment of ESCRT-III induces proliferation of the INM in uninfected cells in a cell cycle-independent manner through ALIX, which is an important adaptor for HSV-1 nuclear egress [80]. Thus, ESCRT-III may control INM integrity by regulating vesicle-mediated cytoplasmic transport in normal cells, although this rate is low and the precise mechanisms that govern this activity are unclear (Figure 4B,D).

The role of ESCRT-III in maintenance of INM integrity may be critical in pathological conditions where INM proteins accumulate abnormally. Hutchinson–Gilford progeria syndrome (HGPS) is s premature aging disorder caused by a point mutation that truncates the LMNA gene [142,143]. In fibroblasts from HGPS patients, truncated LMNA (also referred to as ‘progerin’) accumulates in the nucleus and engenders NM deformations that affect nuclear blebbing and perinuclear vesicle formation [45]. The ALIX-mediated ESCRT-III pathway plays a suppressive role in progerin-induced NM deformation, perhaps through vesicle-mediated cytoplasmic transport [144] (Figure 4B,E). If so, herpesviruses may highjack this system to facilitate nuclear egress of capsids.

## 7. Concluding Remarks

Nuclear egress of viral capsids is essential during the life cycle of herpesviruses. Many groups have extensively studied this mysterious system and uncovered the role of some cellular and viral proteins that are involved in nuclear egress. More recently, the molecular mechanisms that underlie vesicle formation have been revealed by biochemical analysis of the NEC and studies of the cellular scission machinery component, ESCRT-III. In contrast, the precise mechanism of de-envelopment is completely unknown. Moreover, it is not known how and when vesicle formation occurs at the INM in normal cells in the absence of NEC.

Vesicle-mediated nucleocytoplasmic transport might occur rarely and/or be accomplished rapidly in uninfected mammalian cells. If so, it might be difficult to characterize this system without employing exogenous activation or inhibition methods. Studies on the nuclear egress of herpesviruses may help to further elucidate the molecular mechanisms and physiological significance of this transport system in normal situations.

## Figures and Tables

**Table 1:** Viral factors involved in nuclear egress of HSV capsid.

Protein	Conservation	Function
NEC (UL31/UL34)	*Herpesviridae*	Vesicle formation via hexagonal lattice [63]. Incorporation of capsid [13,14]. Dissociation of lamins [29]. Recruitment of ESCRT-III [80]. De-envelopment [81].
UL13	*Herpesviridae*	Dissociation of lamins [82]. (Weak in *Alphaherpesvirinae*).
Us3	*Alphaherpesvirinae*	Dissociation of lamins [33]. Promote de-envelopment [83]. Phosphorylate UL34 [84]. Phosphorylate gB [85,86]. Phosphorylate UL31 [87].
ICP34.5	HSV-1 and HSV-2	Dissociation of lamins via PKC [42].
CVSC (UL17/UL25)	*Herpesviridae*	Link between NEC and capsid [55,56].
UL47	*Alphaherpesvirinae*	Promote primary envelopment [78]. (Not observed in PRV [88,89]).
ICP22	*Alphaherpesvirinae*	Promote primary envelopment [79].
gB and gH/gL	*Herpesviridae*	Redundantly promote de-envelopment [90]. (Not observed in PRV [91]).
UL51	*Herpesviridae*	Promote de-envelopment [92]. (Not observed in PRV [93]).

**Table 2:** Cellular factors involved in nuclear egress of HSV capsids.

Protein	Interactor	Function	*Drosophila* RNP
Lamins	NEC, Us3, UL13	Prevent nuclear egress [29].	Yes [6,122]
PKC family	NEC	Dissociate lamins [32].	Yes [6]
p32	ICP34.5, UL47	Recruit PKC [42]. Promote de-envelopment [116].	-
PKD	-	Promote nuclear egress indirectly [67].	-
VAPB	-	Promote nuclear egress [94].	-
ESCRT-III	NEC	Mediate scission of INM [80].	Yes [80]
ALIX	NEC	Recruit ESCRT-III to INM [80].	-
TSG101	-	Nuclear egress [105].	
CD98hc β1 integrin	NEC, gB, gH	Promote de-envelopment [115]. (Modulate fusion activity?).	-
Torsins	-	Promote de-envelopment indirectly [123].	Yes [124]
